# The Video Collaborative Localization of a Miner’s Lamp Based on Wireless Multimedia Sensor Networks for Underground Coal Mines

**DOI:** 10.3390/s151025103

**Published:** 2015-09-29

**Authors:** Kaiming You, Wei Yang, Ruisong Han

**Affiliations:** School of Electronic and Information Engineering, Beijing Jiaotong University, Beijing 100044, China; E-Mails: youkaiming@bjtu.edu.cn (K.Y.); ruisong@bjtu.edu.cn (R.H.)

**Keywords:** wireless multimedia sensor networks (WMSNs), video collaborative localization, underground coal mine, miner’s lamp

## Abstract

Based on wireless multimedia sensor networks (WMSNs) deployed in an underground coal mine, a miner’s lamp video collaborative localization algorithm was proposed to locate miners in the scene of insufficient illumination and bifurcated structures of underground tunnels. In bifurcation area, several camera nodes are deployed along the longitudinal direction of tunnels, forming a collaborative cluster in wireless way to monitor and locate miners in underground tunnels. Cap-lamps are regarded as the feature of miners in the scene of insufficient illumination of underground tunnels, which means that miners can be identified by detecting their cap-lamps. A miner’s lamp will project mapping points on the imaging plane of collaborative cameras and the coordinates of mapping points are calculated by collaborative cameras. Then, multiple straight lines between the positions of collaborative cameras and their corresponding mapping points are established. To find the three-dimension (3D) coordinate location of the miner’s lamp a least square method is proposed to get the optimal intersection of the multiple straight lines. Tests were carried out both in a corridor and a realistic scenario of underground tunnel, which show that the proposed miner’s lamp video collaborative localization algorithm has good effectiveness, robustness and localization accuracy in real world conditions of underground tunnels.

## 1. Introduction

In order to strengthen production safety, the underground coal mine has an urgent demand for wireless multimedia communication services [1,2,3,4,5]. Wireless multimedia sensor networks (WMSNs) are a new kind of sensor networks that introduce multimedia applications, such as images, and audio and video, into wireless sensor networks (WSNs). It is expected that the multimedia monitoring ability will be improved significantly by applying WMSNs in underground coal mine because of the excellent multimedia perception ability, fast and convenient wireless access and flexible topology characteristics of WMSNs [6,7,8,9,10]. In this article, WMSNs in an underground coal mine are structured and a video collaborative localization algorithm for miner is proposed based on the WMSNs to improve the safety of miners.

In WMSNs, video localization of targets is divided into localization with a single camera [11,12] and localization with collaborative cameras [13,14,15,16,17,18,19,20]. It is usually required to obtain the height of target in advance [11] or to extract target perfectly [12] for localization with single camera. Since the altitude localization and plane localization of miner’s lamp need to be detected simultaneously, the localization with collaborative cameras are applied in this paper. Video collaborative localization of targets is achieved through fusing the video information collected and processed by multiple cameras. For example, reference [13] proposed a visual collaborative localization algorithm for the passive target positioning problem. According to machine vision theory, the target was projected into several straight lines on the ground plane. The optimum intersection of straight lines, which could be obtained by using Hough transform, was the position estimation of the target. However, because of narrow structures of underground tunnels, cameras can only be deployed along the longitudinal direction of tunnels. Thus, the overlap of projection straight lines may result in location failure with this method.

References [14,15] proposed a method to utilize the active cameras to localize the target collaboratively. The perspective projection model of target was established by utilizing the Gaussian error function. Cameras identify the target location by recognizing the target body and calculating the position and the size of target body based on the perspective projection model. Moreover, the problem of active cameras selection was tackled by balancing the tradeoff between the accuracy of target localization and the energy consumption in camera sensor networks. 

Reference [16] presented a technique of multiple feature points to compute the target location in the wireless visual sensor networks. The target edge was determined by Canny detector and the position of target was calculated by fusing the edge information of the target. However, since underground coal mine has feature of insufficient illumination and there may be a lot of coal dusts attaching on miners’ uniforms [21], miner’s body and the mine’s background are very similar, which makes it difficult to distinguish miner’s profile from the mine’s background.

Therefore, considering insufficient illumination and narrow underground tunnels with bifurcated structures, a miner’s lamp video collaborative localization algorithm based on WMSNs was proposed. Since miners must carry cap-lamps to offer auxiliary lighting in underground coal mine which provide a sharp contrast to the dim background, in the algorithm, it was proposed to locate a miner by detecting the cap-lamp of the miner. The miner’s lamp project mapping points on the imaging plane of cameras and the coordinates of mapping points are calculated by cameras. Then, multiple straight lines between the positions of cameras and their corresponding mapping points are established. Finally, a miner can be located by finding the optimal intersection of the multiple straight lines. To verify the effectiveness of the proposed miner’s lamp video collaborative localization algorithm, tests were carried out both in a corridor and a realistic scenario of underground tunnel. The experimental results show that good localization performance is achieved with the proposed algorithm.

The rest of this article is organized as follows. Section 2 introduces the architecture of WMSNs and location scene for underground coal mine. The algorithm of video collaborative localization of the miner’s lamp is discussed in Section 3 and Section 4 presents the experiments results. Section 5 concludes the article.

## 2. Architecture of WMSNs and Location Scene for Underground Coal Mine

**Figure 1:**
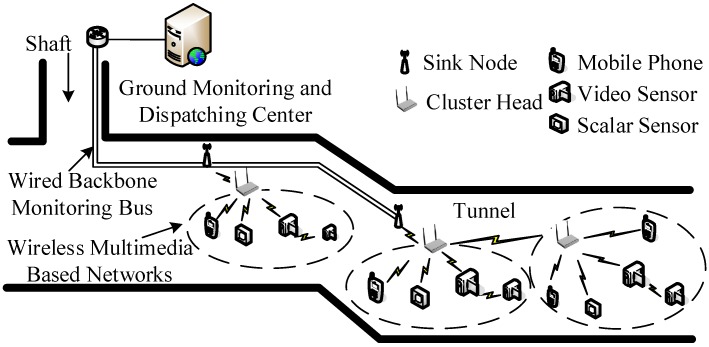
Architecture of wireless multimedia sensor networks (WMSNs) in underground coal mine.

Figure 1 shows the architecture of WMSNs in underground coal mine. As shown in Figure 1, the system consists of three layers, *i.e*., wireless multimedia based networks, wired backbone monitoring bus as well as Ground Monitoring and Dispatching Center (GMDC). The wireless multimedia based networks are constituted by cameras, mobile phones and scalar sensors, *etc*. They are deployed in underground coal mine to collect multimedia information. The multimedia information collected is processed by cluster head nodes and sent to the sink nodes in multi-hop relaying way. Sink nodes which connect with wired backbone monitoring bus transmit multimedia information collected to GMDC via wired backbone monitoring bus. In this way, the GMDC can monitor the multimedia information of underground coal mine in real time.

**Figure 2:**
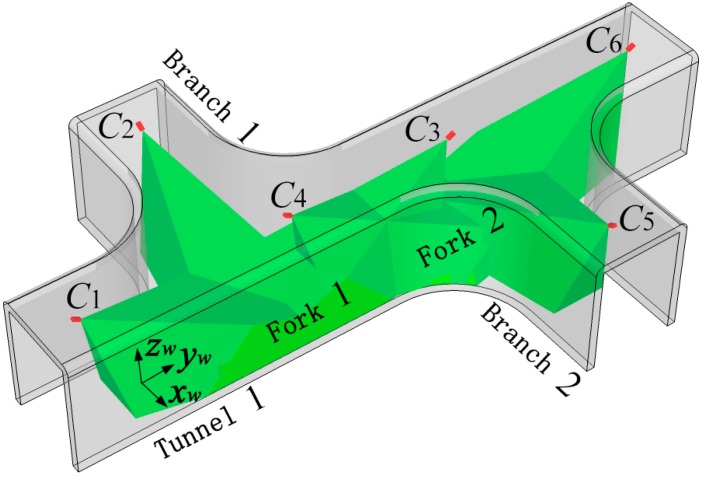
Scene of miner localization in underground coal mine with bifurcated structures.

Figure 2 shows the scene of miner localization in underground coal mine with bifurcated structures. With the purpose of describing the positions of cameras and miners, a unified World Coordinate System (WCS) *o_w_x_w_y_w_z_w_* is established along underground tunnels with the transverse direction of the Tunnel 1 as *x_w_*-axis, the longitudinal direction as *y_w_*-axis and the altitude direction as *z_w_*-axis, respectively. The tunnel entrance is selected as the origin of the WCS, so that any point in underground tunnels can be located according to its position along the tunnels. It notes that any underground tunnels can be described with the WCS.

In order to locate miners, the WMSNs cameras deployed in underground tunnels should be aware of positions of themselves as well as positions of adjacent cameras which are referred to the geometrical parameters in this paper. In fact, several algorithms of localization and calibration can be applied to obtain the geometrical parameters automatically [22,23,24,25,26]. However, in our experiments, cameras are deployed manually and their geometrical parameters are measured also manually.

The gray part in Figure 2 represents underground tunnels with bifurcated structures. Tunnel 1 and Branch 1, Branch 2 constitute the bifurcation areas of Forks 1 and 2. *C_i_* in Figure 2 represents camera nodes which have the same image processing, digital processing, wireless transmission and reception properties. Cameras *C*_1_, *C*_2_ and *C*_3_ are deployed along the longitudinal direction of tunnels, facing Fork 1 and monitor Fork 1 together. Similarly, cameras *C*_4_, *C*_5_ and *C*_6_ are deployed along the longitudinal direction of tunnels, facing Fork 2 and monitor Fork 2 together. Cameras *C*_3_ and *C*_4_ are deployed along the longitudinal direction of Tunnel 1, monitoring the connected region of Fork 1 and Fork 2 together. The green shadow part in Figure 2 represents the areas that cameras monitor. The area that one camera can monitor is approximately a pyramid shape.

## 3. Video Collaborative Localization of the Miner’s Lamp

### 3.1. The Miner’s Lamp Detection with the Background Subtraction Method

In underground tunnels, generally it is difficult to distinguish miners because of coal dusts and insufficient illumination. However, miners must carry cap-lamps to offer auxiliary lighting in the underground coal mine, which provide a sharp contrast to the dim background. Thus, the background subtraction method [27] is applied in order to detect the miners’ lamps reliably.

From the collected video sequences of underground tunnels, we select one frame image without miner as the initial background image. The difference image is obtained through a subtraction between the current image and the background image. In order to reduce the noise interference caused by the beam divergence of miner’s lamp, the mean filter is applied to smooth the difference image obtained. An appropriate threshold is selected to turn filtered difference image into binary image which has only two pixel value, 0 and 1.

If pixel value of the binary image obtained is 0, it indicates that there is no miner in the monitoring area of a camera. Then, the background image is updated with the current image of video sequences collected by camera at the top of tunnel. In contrast, if any of the pixel values of the binary image is 1, it indicates that a miner has appeared in the monitoring area of a camera. The mapping range on the imaging plane of camera is the region with pixel value of binary image being 1, which indicates that there is a miner with cap-lamp in the area. The pixel coordinate of the mapping point of the miner’s lamp on the imaging plane of camera can be obtained by calculating the geometric center of the mapping range. 

### 3.2. Camera Imaging Model

**Figure 3:**
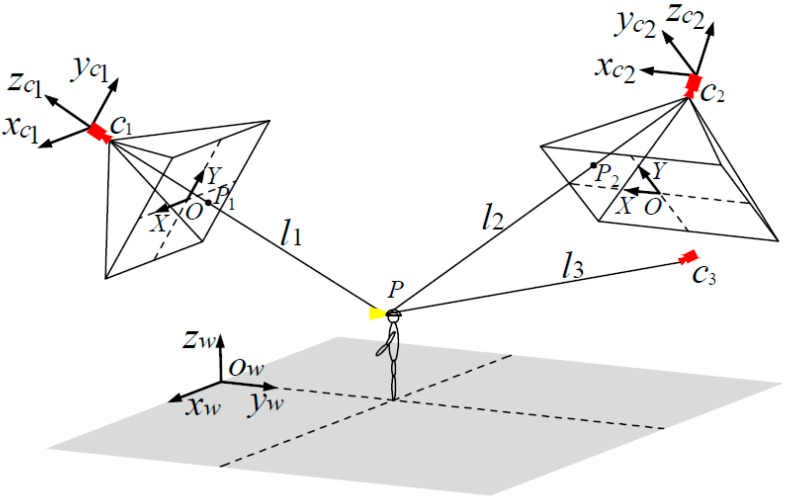
Camera vision imaging model.

The pixel coordinate of the mapping point needs to be converted to the physical coordinate in the WCS of underground coal mines. Thus, a vision imaging model of cameras is established according to the linear imaging principle of the camera [28]. Figure 3 shows the vision imaging model of cameras *C*_1_, *C*_2_ and *C*_3_ in Figure 2.

The grey shade of Figure 3 represents the ground part of tunnels in Figure 2. *P* is the position where a miner carries a cap-lamp. *C_i_* is the camera node, assuming its position coordinate in the WCS of underground coal mine is *C_i_*(*x_wci_*,*y_wci_*,*z_wci_*), where the value of *i* is 1, 2 and 3. The *o_w_x_w_y_w_z_w_* in Figure 3 represents the WCS of underground coal mine that has been established in Figure 2. *OXY* represents the two-dimensional coordinate system of the imaging plane of camera. *X* axis and *Y* axis are respectively the horizontal and vertical direction on the imaging plane of camera. The pyramid composed of camera and its imaging plane represents the region that a camera covers. The Camera Coordinate System (CCS) *o_ci_x_ci_y_ci_z_ci_* of *C_i_* is established, where the position of *C_i_* is selected as the origin of its CCS, *z_ci_* axis is the opposite direction of optical axis of the camera, *x_ci_* axis and *y_ci_* axis are respectively same with the directions of *X* axis and *Y* axis on the imaging plane of camera.

In Figure 3, we assume that the camera *C_i_* detects a miner’s lamp and calculates the pixel coordinate *P_i_*(*u_i_*,*v_i_*) of the mapping point on its own imaging plane. By applying the linear relationship between image resolution of the camera *C_i_* and physical size of the photosensitive element Complementary Metal Oxide Semiconductor (CMOS) in the Camera Coordinate System *o_ci_x_ci_y_ci_z_ci_*, the pixel coordinate *P_i_* (*u_i_*,*v_i_*) of the mapping point is converted to the physical coordinate *P_ci_*(*x_ci_*,*y_ci_*,*z_ci_*) in the CCS *o_ci_x_ci_y_ci_z_ci_* as

(1) $$\left[\begin{array}{c} x_{ci}\\ y_{ci}\\ z_{ci} \end{array}\right]=\left[\begin{array}{ccc} m/M & 0 & -m/2\\ 0 & -n/N & n/2\\ 0 & 0 & -f \end{array}\right]\left[\begin{array}{c} u_{i}\\ v_{i}\\ 1 \end{array}\right]$$

where *f* is the focal length of camera *C_i_*, *m* and *n* are the physical dimensions of the photosensitive element CMOS in the horizontal and vertical direction. The image resolution is *M* × *N*, where *M* and *N* are respectively the number of pixels in the *X* axis and *Y* axis direction on the imaging plane. Expressions *m*/*M* and *n*/*N* are respectively the physical size of a single pixel in the *X* axis and *Y* axis direction on the imaging plane of camera.

The physical coordinate *P_ci_*(*x_ci_*,*y_ci_*,*z_ci_*) of the mapping point in the CCS *o_ci_x_ci_y_ci_z_ci_* need to be converted to the physical coordinate in the WCS *o_w_x_w_y_w_z_w_*. We assume that the CCS *o_ci_x_ci_y_ci_z_ci_* is overlapped with the WCS *o_w_x_w_y_w_z_w_* when the WCS *o_w_x_w_y_w_z_w_* rotate *φ* degrees counterclockwise around *z_w_* axis, rotate *θ* degrees counterclockwise around *x_w_* axis and translate a vector *t_wi_* in turn. Then, the physical coordinate *P_ci_*(*x_ci_*,*y_ci_*,*z_ci_*) in the CCS *o_ci_x_ci_y_ci_z_ci_* is turned into the physical coordinate *P_wi_*(*x_wi_*,*y_wi_*,*z_wi_*) in the WCS *o_w_x_w_y_w_z_w_* as

(2) $$\left[\begin{array}{c} x_{wi}\\ y_{wi}\\ z_{wi}\\ 1 \end{array}\right]=\left[\begin{array}{cc} R_{wi} & t_{wi}\\ 0_{3}^{T} & 1 \end{array}\right]\left[\begin{array}{c} x_{ci}\\ y_{ci}\\ z_{ci}\\ 1 \end{array}\right]$$

where the translational vector *t_wi_* = (*x_wci_*,*y_wci_*,*z_wci_*) is from the origin point to the camera *C_i_* node in the WCS of underground coal mine. Rotation matrix *R_wi_* is decided by the yaw angle *φ* and the pitch angle *θ* of the camera *C_i_* as

(3) $$R_{wi}=\left[\begin{array}{ccc} \cos\varphi & -\sin\varphi \cos\theta & \sin\varphi \sin\theta \\ \sin\varphi & cos\varphi \cos\theta & -cos\varphi \sin\theta \\ 0 & \sin\theta & \cos\theta \end{array}\right]$$

### 3.3. Miner’s Lamp Video Collaborative Localization

When a miner enters the bifurcation area of tunnels where several camera nodes have been deployed, camera *C_i_* detects the miner’s lamp and calculates its pixel coordinate *P_i_*(*u_i_*,*v_i_*). Assume that cameras which detect the miner’s lamp form a cluster by themselves in wireless way and they are in a state of time synchronization. Transforming the pixel coordinate *P_i_*(*u_i_*,*v_i_*) with the Equation (1), the physical coordinate *P_ci_*(*x_ci_*,*y_ci_*,*z_ci_*) of a miner’s lamp mapping point in the CCS *o_ci_x_ci_y_ci_z_ci_* can be obtained. Therefore, the physical coordinate *P_wi_*(*x_wi_*,*y_wi_*,*z_wi_*) of a miner’s lamp mapping point in the WCS of underground coal mine can also be obtained by applying the Equation (2) to the physical coordinate *P_ci_*(*x_ci_*,*y_ci_*,*z_ci_*).

Establish a straight line *l_i_* between the position coordinate of camera *C_i_*(*x_wci_*,*y_wci_*,*z_wci_*) and the physical coordinate *P_wi_*(*x_wi_*,*y_wi_*,*z_wi_*) as

(4) $$\frac{x_{l\text{i}}-x_{wi}}{x_{wi}-x_{wci}}=\frac{y_{li}-y_{wi}}{y_{wi}-y_{wci}}=\frac{z_{li}-z_{wi}}{z_{wi}-z_{wci}}=s_{i}$$

where (*x_li_*,*y_li_*,*z_li_*) is the coordinate of the point on the straight line *l_i_* in the WCS and *s_i_* is the parameter of the straight line *l_i._*

The three-dimension (3D) position coordinate of a miner’s lamp in the WCS can be obtained by calculating the intersection of straight lines between the positions of cameras and their corresponding mapping points. However, the underground tunnels are complex and errors of camera measurement caused by position coordinate, yaw angle and pitch angle cannot be avoided. In addition, miner’s lamp orientation with respect to different cameras may be different, which may lead to identification errors of miner’s lamp mapping points because of the beam divergence of miner’s lamp. As a result, multiple straight lines *l_i_* between the positions of cameras and their corresponding mapping points generally cannot intersect at one point.

We solve this problem by finding an optimal intersection *P* in the WCS who achieve the minimum square sum *J* of the distance to multiple straight lines *l_i_* as

(5) $$\{\begin{array}{c} P=\arg\min(J),J=\sum d_{i}^{2}\\ d_{i}^{2}={\left(x-x_{li}\right)}^{2}+{\left(y-y_{li}\right)}^{2}+{\left(z-z_{li}\right)}^{2} \end{array}$$

The extremum problem of Equation (5) can be solved by calculating the partial derivative of *x*, *y*, *z* and *s_i_* respectively and make its value be equal to 0 as

(6) $$\frac{\partial J}{\partial x}=0,\frac{\partial J}{\partial y}=0,\frac{\partial J}{\partial z}=0,\frac{\partial J}{\partial s_{i}}=0$$

By this way, we can get the least square estimation of the optimal intersection *P* which provides the position coordinate of miner’s lamp in the WCS of underground coal mine. According to the 3D coordinate of a miner’s lamp, the plane position coordinate (*x*,*y*) of the miner in a tunnel and the altitude coordinate *z* of the miner’s lamp to the ground can also be obtained.

## 4. Experiments and Performances

### 4.1. Experiment in a Corridor

**Figure 4:**
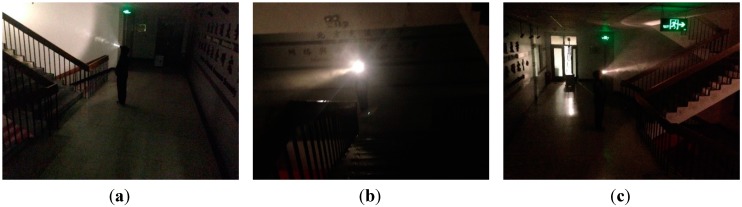
(**a**) The image of the corridor taken by camera *C*_1_; (**b**) The image of the corridor taken by camera *C*_2_; (**c**) The image of the corridor taken by camera *C*_3_.

In order to evaluate the performances of the proposed miner’s lamp video collaborative localization algorithm based on underground WMSNs, a test was carried out. A corridor with stairs shown in Figure 4 was selected to simulate the bifurcation structure of underground tunnels. Lights in the corridor were turned off to simulate the insufficient illumination tunnels. Smoke machine sprayed smoke in the corridor to simulate tunnels with great amounts of water vapor and dusts. The tester wore dark blue overalls and safety helmet with miner’s lamp turning on to simulate miners in underground tunnels. A World Coordinate System (WCS) was established at the entrance of the corridor with the transverse direction of the corridor as *x_w_*-axis, the longitudinal direction as *y_w_*-axis and the altitude direction as *z_w_*-axis, respectively. 

**Figure 5:**
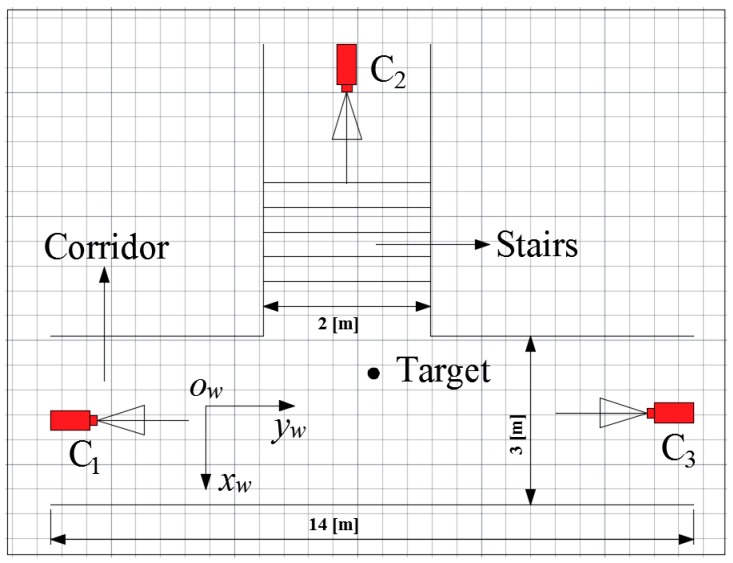
Topology of the experiment.

In the test, three isomorphic cameras TY803-130 were adopted manually at the top of the corridor by tripods. The cameras *C*_1_, *C*_2_ and *C*_3_ were deployed according to Figure 2, and the topology of the experiment is shown in Figure 5. Cameras are wirelessly linked by Wi-Fi with carrier frequency 2.4 GHz and 802.11 n transmission protocol. Table 1 lists the intrinsic parameters of three cameras, including focal length *f*, image resolution *M* × *N* and the physical size of the photosensitive element CMOS. Table 2 lists the geometrical parameters of three cameras, including the world coordinates of the locations of cameras, the angle of yaw *φ* between the WCS *x_w_*-axis and the Camera Coordinate System (CCS) *x_w_*-axis in counterclockwise direction and the *θ* between the WCS *z_w_*-axis and the CCS *z_w_*-axis in counterclockwise direction.

**Table 1:** Intrinsic parameters of cameras.

Parameter	Value
*f*/mm	6.0
*M* × *N* /pixel	1280 × 960
*CMOS*/mm	4.8 × 3.6

**Table 2:** Geometrical parameters of cameras.

	(*x_w_*,*y_w_*,*z_w_*)/m	*φ*/°	*θ*/°
Camera 1	(0.29, −2.20, 2.32)	0	70
Camera 2	(−6.33, 2.86, 3.08)	−90	64
Camera 3	(0.15, 8.95, 2.34)	180	70

The tester walked into the corridor at a normal pace. Cameras *C*_1_, *C*_2_ and *C*_3_ worked together to monitor the corridor and collected the video information at the same time. At a certain time point, cameras *C*_1_, *C*_2_ and *C*_3_ collected the images of corridor, as is shown in Figure 4a–c, respectively.

Cameras *C*_1_, *C*_2_ and *C*_3_ detected the miner’s lamp by the difference value between the current images from their video sequences and the background images. In the experiment, 60% maximum grey value of difference image is selected as threshold. Cameras *C*_1_, *C*_2_ and *C*_3_ detected the miner’s lamp at interval of 0.5 s and calculated the pixel coordinates of mapping points of the miner’s lamp on their own imaging plane. Then, the pixel coordinates were translated to the coordinates in the WCS by applying Equations (1) and (2). Therefore, three straight lines of three-dimension (3-D) between cameras *C*_1_, *C*_2_ and *C*_3_ and their corresponding mapping points of the miner’s lamp could be established in the WCS.

By applying Equations (5) and (6) to calculate the optimum intersection of the three straight lines, the position coordinate of three-dimension (3-D) of the miner’s lamp in the WCS could be obtained which also provided both the plane coordinate of the tester in the corridor and the altitude of the miner’s lamp from the corridor ground. 10 gauge points on the ground of the corridor had been marked and measured to show the localization precision of the miner’s lamp. The tester would walk to pass these 10 gauge points and stay for one second at each gauge point. By applying the proposed algorithm, cameras *C*_1_, *C*_2_ and *C*_3_ could locate the tester’s lamp automatically at interval of 0.5 s when the tester passed each gauge point. 

In order to compare the estimated localization of the tester’s lamp with the actual location of the tester’s lamp, the error of altitude localization is defined as

(7) $$E_{h}(i)=\left|h_{r}(i)-h_{l}(i)\right|$$

where *h_r_*(*i*) is the actual altitude of the tester’s lamp from the tunnel ground and *h_l_*(*i*) is the altitude of the tester’s lamp obtained by the proposed algorithm. Thus, the average error of the altitude localization is defined as

(8) $${\bar{E}}_{h}=\frac{1}{N}\sum_{i=1}^{N}E_{h}(i)$$

where *N* is the number of gauge points in the test.

Similarly, the error of plane localization which includes errors both in *x*-axis and *y*-axis is defined as

(9) $$E_{P}(i)=\sqrt{{\left[x_{r}(i)-x_{l}(i)\right]}^{2}+{\left[y_{r}(i)-y_{l}(i)\right]}^{2}}$$

where $\left(x_{r}(i),y_{r}(i)\right)$ is the actual plane location of the tester’s lamp and $\left(x_{l}(i),y_{l}(i)\right)$ is the estimated plane coordinates with the proposed algorithm. Thus, the average error of the plane localization is defined as

(10) $${\bar{E}}_{p}=\frac{1}{N}\sum_{i=1}^{N}E_{p}(i)$$

where *N* is the number of gauge points in the test.

**Figure 6:**
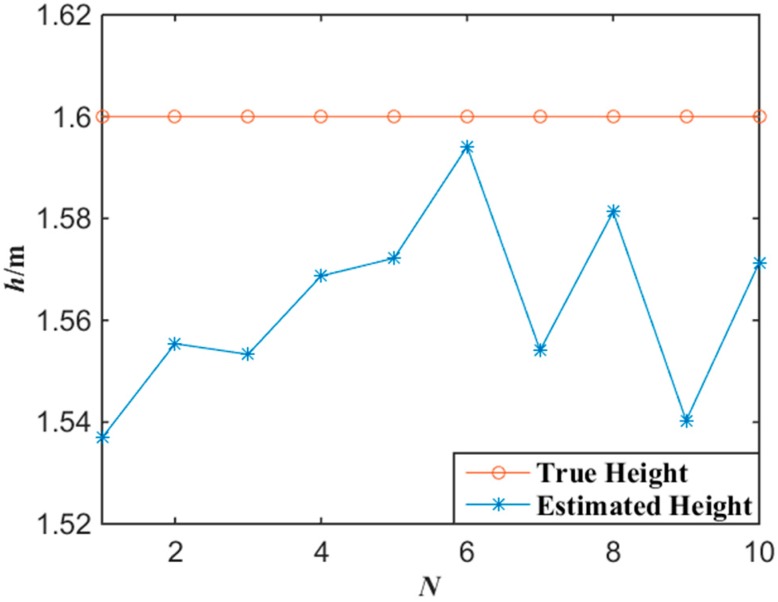
Experimental data of altitude with the miner’s lamp video collaborative localization.

Figure 6 shows the experimental results of the altitude localization of tester’s lamp with the proposed algorithm. According to the experimental data of Figure 6, the average error of the altitude localization of the tester is 3.7 cm by applying Equations (7) and (8). 

**Figure 7:**
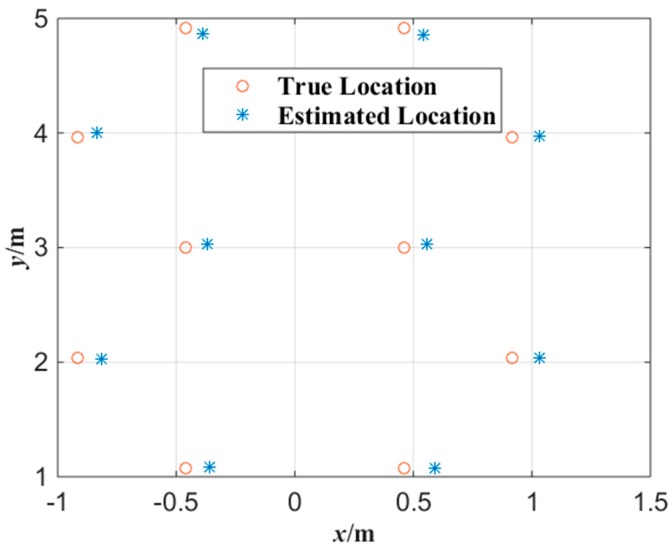
Experimental data of plane localization with the miner’s lamp video collaborative localization.

**Figure 8:**
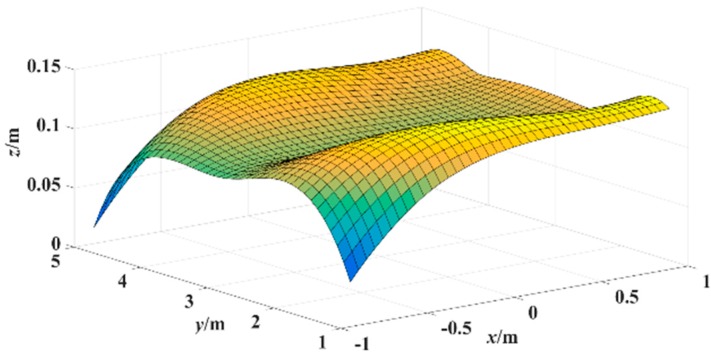
Error surface of plane localization with the miner’s lamp video collaborative localization.

Figure 7 shows the experimental results of the plane localization of tester’s lamp with the proposed algorithm where the tester stood at each gauge point passed. According to the experimental data of Figure 7, the error surface of plane localization is shown in Figure 8 which applies a linear interpolation algorithm to the error of plane localization calculated with Equation (9). In Figure 8, *x-y* plane is the corridor ground, (*x*,*y*) coordinate represents the tester’s actual location, and coordinate *z* represents the error of plane localization between the locations estimated and the actual locations. As shown in Figure 8, the average error of the plane localization is around 10 cm with the proposed algorithm.

**Figure 9:**
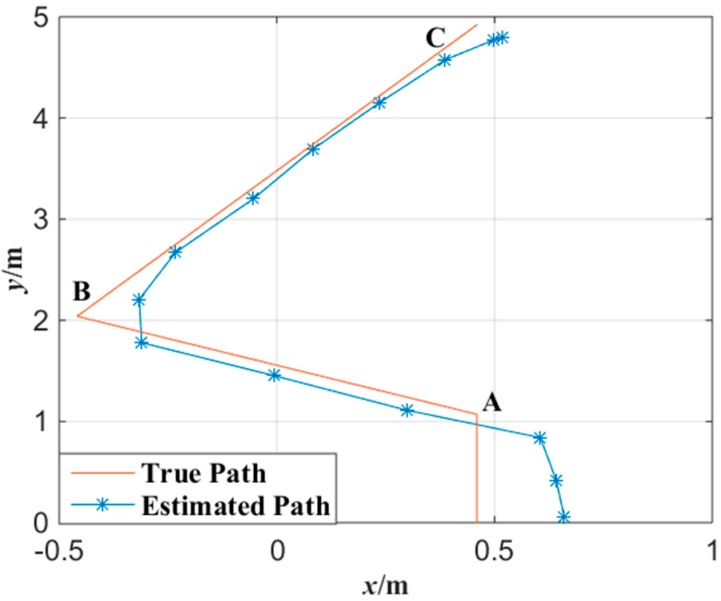
Trajectory tracked with the miner’s lamp video collaborative localization.

In order to evaluate the performance of the miner’s lamp collaborative localization algorithm under the condition that the miner moves continuously, cameras *C*_1_, *C*_2_ and *C*_3_ were also applied to detect the movement of the tester in the corridor. The tester walked straight into the corridor from the entrance at a normal pace. Then, the tester turned about 45° at point A and walked about 1.5 m to point B. After that, the tester turned again at point B and walked toward point C. Figure 9 shows the tester’s actual motion trajectory as well as the estimated motion trajectory with the proposed algorithm at interval of 0.5 s. In Figure 9, the motion trajectory estimated is obtained by connecting two adjacent tester’s locations with line segments.

In the bottom right of Figure 9, it can be observed that the location error is larger when the tester began to enter into the corridor. This is because when the tester began to enter into the corridor, the mapping points of tester’s cap-lamp were respectively on the edge of the imaging planes of cameras *C*_1_, *C*_2_ and *C*_3_ at the same time. Due to the non-linear relationship between object and image on the imaging edge [29], the mapping points of tester’s cap-lamp on the imaging planes will distort to some extent which will lead to larger location error. However, the maximum error for the movement orbit tracing is about 20 cm which is also acceptable to trace a miner. In contrast, the location error is smaller when the tester was around central area of the corridor. This is because the images of cameras *C*_1_, *C*_2_ and *C*_3_ have a good linear relationship when the miner’s lamp is around central area which results in smaller errors. 

### 4.2. Experiment in an Underground Tunnel

**Figure 10:**
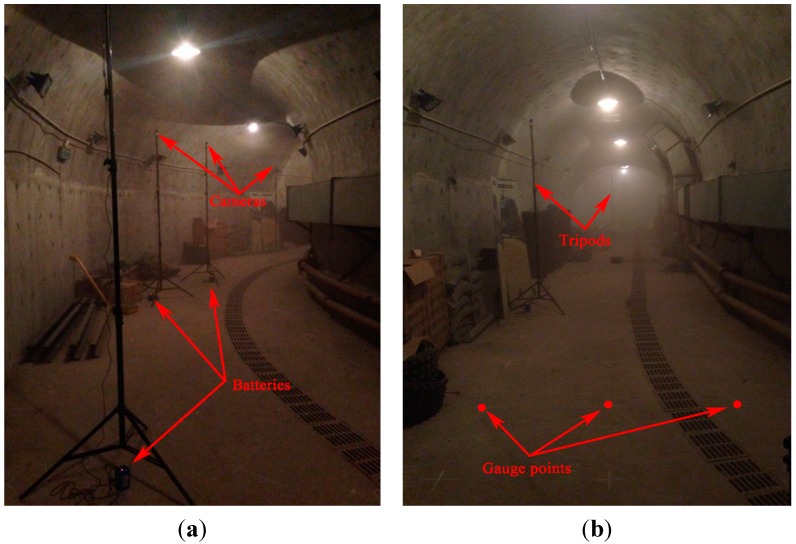
(**a**) The actual deployment of cameras; (**b**) Gauge points on the ground of the tunnel.

In order to evaluate the performances of the proposed algorithm in the realistic scenario of underground tunnels, a test was also carried out in the realistic scenario of an underground tunnel. The test was performed in the underground tunnel of Track Vibration Abatement and Control Laboratory at Beijing Jiaotong University, as shown in Figure 10. The width and the altitude of the underground tunnel are 4 m with horseshoe shaped structure. In the test, six isomorphic cameras TY803-130 were deployed manually at the top of the tunnel by tripods. The cameras *C*_1_, *C*_2_, *C*_3_, *C*_4_, *C*_5_ and *C*_6_ were powered by lithium batteries.

**Figure 11:**
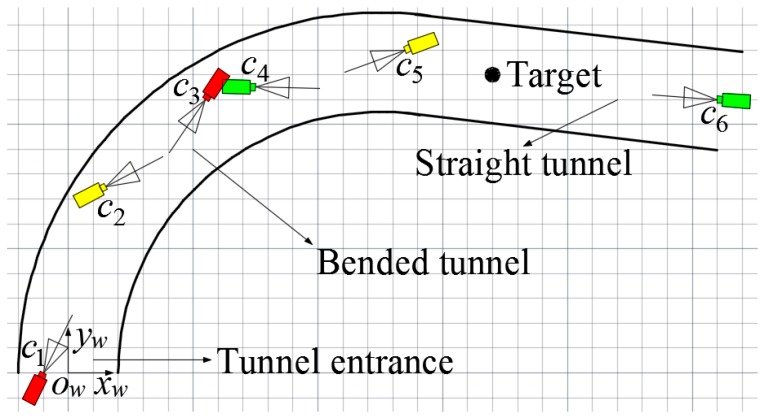
Topology of the experiment.

Figure 10 and Figure 11 show the actual deployment of cameras and the topology of the experiment, respectively. A World Coordinate System (WCS) was established at the entrance of the tunnel with the transverse direction of the tunnel as *x_w_*-axis, the longitudinal direction as *y_w_*-axis and the altitude direction as *z_w_*-axis, respectively. As shown in Figure 11, the six isomorphic cameras were divided into three groups and the two cameras in the same group were deployed oppositely, with cameras *C*_1_ and *C*_3_ as the first group, cameras *C*_2_ and *C*_5_ as the second group and cameras *C*_4_ and *C*_6_ as the third group. Cameras *C*_1_ and *C*_3_ are deployed crosswise along the direction of center line of the bended tunnel to monitor the area of tunnel entrance together. In the same way, cameras *C*_2_ and *C*_5_ are deployed crosswise along the direction of center line of the bended tunnel to monitor the area of bended tunnel together. Similarly, cameras *C*_4_ and *C*_6_ are deployed crosswise along the longitudinal direction of the straight tunnel to monitor the area of straight tunnel together.

The intrinsic parameters of six cameras, including focal length *f*, image resolution *M* × *N* and the physical size of the photosensitive element CMOS are listed in Table 1. Table 3 lists the geometrical parameters of six cameras, including the world coordinates of the locations of cameras, the angle of yaw *φ* between the WCS *x_w_*-axis and the CCS *x_w_*-axis in counterclockwise direction and the *θ* between the WCS *z_w_*-axis and the CCS *z_w_*-axis in counterclockwise direction.

**Table 3:** Geometrical parameters of cameras.

	(*x_w_*,*y_w_*,*z_w_*)/m	*φ*/°	*θ*/°
Camera 1	(−1.00, 0, 2.87)	−26.6	68.0
Camera 2	(1.50,7.50, 2.90)	−62.7	67.4
Camera 3	(5.50, 11.00, 2.88)	146.1	70.6
Camera 4	(7.50, 11.50, 2.90)	−91.2	71.8
Camera 5	(13.50, 13.00, 2.87)	110.1	71.2
Camera 6	(26.00, 11.00, 2.85)	86.0	72.1

**Figure 12:**
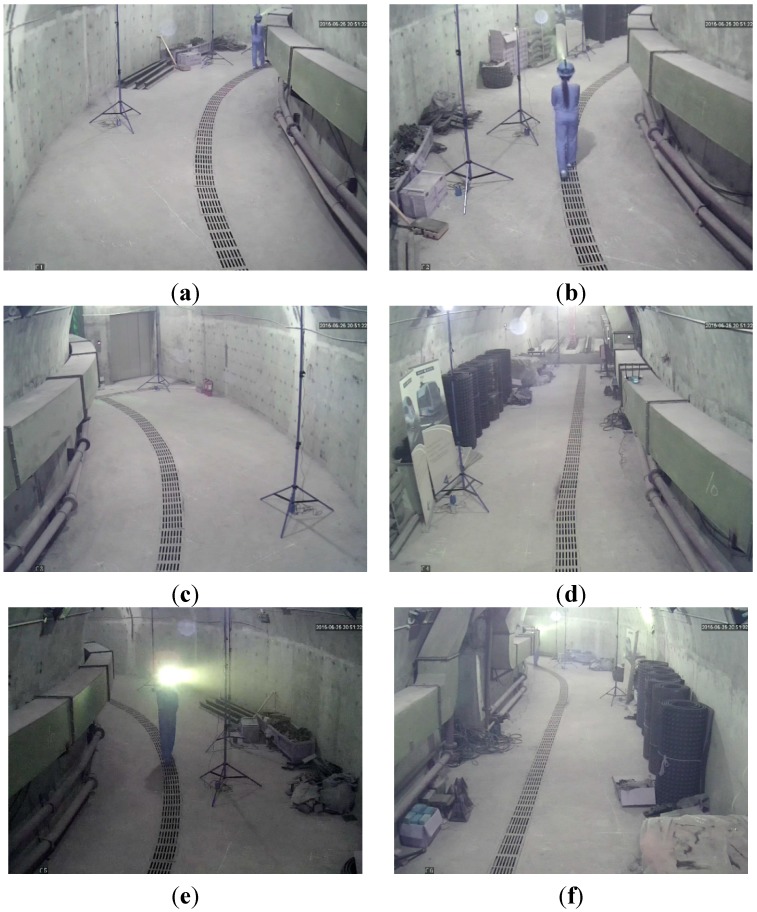
(**a**) The image of the tunnel taken by camera *C*_1_; (**b**) The image of the tunnel taken by camera *C*_2_; (**c**) The image of the tunnel taken by camera *C*_3_; (**d**) The image of the tunnel taken by camera *C*_4_; (**e**) The image of the tunnel taken by camera *C*_5_; (**f**) The image of the tunnel taken by camera *C*_6_.

In the test, tester wore dark blue overalls and safety helmet with miner’s lamp turning on to simulate miners in underground tunnels. The tester walked into the tunnel at a normal pace. Cameras *C*_1_, *C*_2_, *C*_3_, *C*_4_, *C*_5_ and *C*_6_ worked together to monitor the tunnel and collected the video information at the same time. Figure 12a–f showed the images of the tunnel collected by cameras *C*_1_, *C*_2_, *C*_3_, *C*_4_, *C*_5_ and *C*_6_ at a certain time point, respectively.

Cameras *C*_1_ and *C*_3_, cameras *C*_2_ and *C*_5_, and cameras *C*_4_ and *C*_6_ detected the miner’s lamp by the difference value between the current images from their video sequences and the background images, respectively. In the experiment, 60% maximum grey value of difference image is selected as threshold. Cameras *C*_1_, *C*_2_, *C*_3_, *C*_4_, *C*_5_ and *C*_6_ detected the miner’s lamp at interval of 0.5 s and calculated the pixel coordinates of mapping points of the miner’s lamp on their own imaging plane. Then, the pixel coordinates were translated to the coordinates in the WCS by applying Equations (1) and (2).

Therefore, when two cameras of a certain group detected the miner’s lamp simultaneously, two straight lines of three-dimension (3-D) between two cameras in the group and their corresponding mapping points of the miner’s lamp could be established in the WCS. By applying Equations (5) and (6) to calculate the optimum intersection of the two straight lines, the position coordinate of three-dimension (3-D) of the miner’s lamp in the WCS could be obtained which provided both the plane coordinate of the tester along the tunnel and the altitude of the miner’s lamp from the tunnel ground.

In order to evaluate the localization precision of the proposed algorithm when applied to the realistic scenario of underground tunnel, dozens of gauge points had been marked on the ground of the tunnel, as shown in Figure 10b. Gauge points formed grids with horizontal and vertical spacing of one meter (1 m) on the ground of the tunnel.

In the test, the tester walked and passed these gauge points. The tester would stand and squat about one second (1 s) at each gauge point passed, respectively. The coordinates of gauge points in the WCS and the altitude of the miner’s lamp from the tunnel ground when the tester stood and squatted had been measured manually which provided actual locations of tester’s lamp. When the tester could be located by cameras of two groups simultaneously, the average value of localizations of the two groups was selected as the localization result of the tester’s lamp.

**Figure 13:**
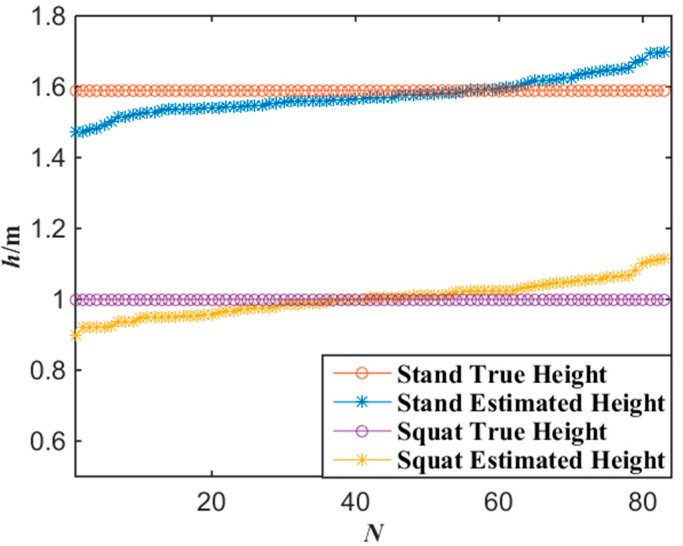
Experimental data of altitude with the miner’s lamp video collaborative localization.

Figure 13 shows the experimental results of the altitude localization of tester’s lamp with the proposed algorithm where the tester stood and squatted at each gauge point passed, respectively. According to the experimental data of Figure 13, when the number of gauge points is 83, the average error of the altitude localization for up-right and squat postures of the tester are 4.7 cm and 3.9 cm by applying Equations (7) and (8), respectively. Generally, when the error of altitude localization is less than 10 cm, the miners’ posture of standing or squatting can be correctly distinguished. Thus, 10 cm error of altitude localization can be accepted. If the altitude of a miner’s lamp is similar to a normal human altitude, it indicates that the miner is in the posture of walking or standing. However, if the altitude of a miner’s lamp is much lower than normal human altitude, it indicates that the miner may be in other postures, for example, in the posture of squat. No matter what posture a miner keeps, the altitude of a miner’s lamp can be obtained with the proposed miner’s lamp video collaborative localization algorithm based on WMSNs and the posture of the miner can be analyzed.

**Figure 14:**
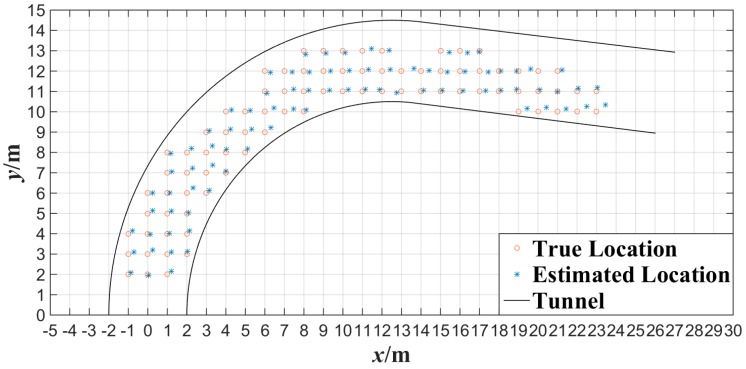
Experimental data of plane localization with the miner’s lamp video collaborative localization.

**Figure 15:**
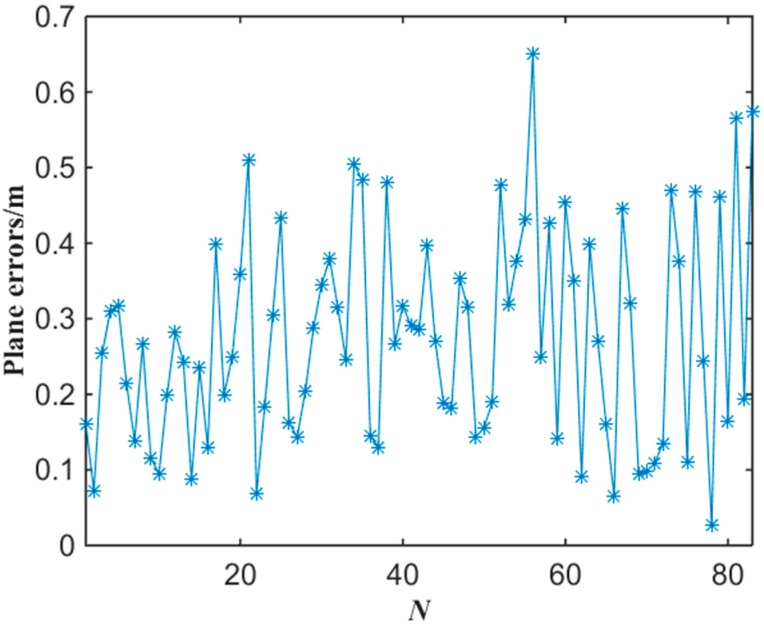
The error of plane localization with the miner’s lamp video collaborative localization.

Figure 14 shows the experimental results of the plane localization of tester’s lamp with the proposed algorithm where the tester stood at each gauge point passed. Note that the intersection of grids in Figure 14 is the gauge point with 1 m spacing. We can note that most of the location points estimated have errors in the same direction compared with the gauge points in Figure 14. This is mainly because that the tester’s lamp was always toward the opposite direction of tunnel entrance while walking in the test. According to the experimental data of Figure 14, the error of plane localization for up-right posture of the tester can be obtained by applying Equation (9) as shown in Figure 15. When the number of gauge points is 83, the average error of the plane localization for up-right posture of the tester is 27.4 cm by applying Equation (10). Similarly, when the error of plane localization is less than 50 cm, there will be no obvious deviation for the estimation of plane localization or trajectory of miners along tunnels. The experimental results of Figure 14 and Figure 15 indicate that the plane coordinates of the tester on the ground of a tunnel can be precisely estimated with the proposed miner’s lamp video collaborative localization algorithm. It is worth mentioning that it can be ensured to recognize a miner’s lamp by adjusting the threshold effectively. However, since the illumination of miner’s lamp is generally intensive in different background illumination such as in Figure 4 and Figure 10, 60% maximum grey value of difference image was selected as the threshold, which provided a good performance of recognition and localization accuracy in both scenarios.

**Figure 16:**
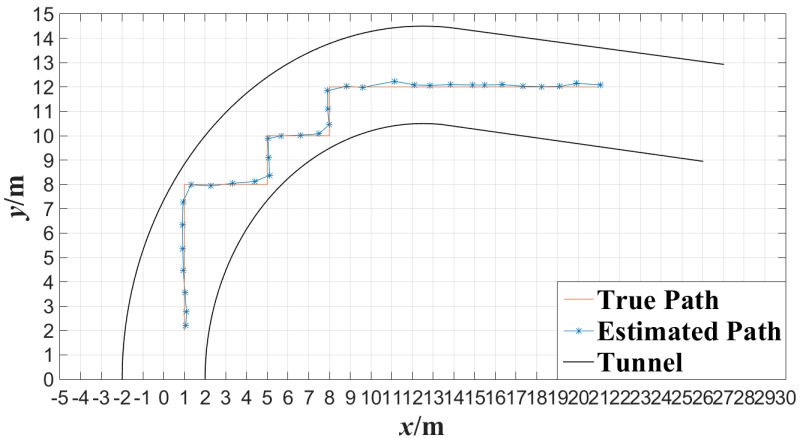
Trajectory of broken line tracked with the miner’s lamp video collaborative localization.

**Figure 17:**
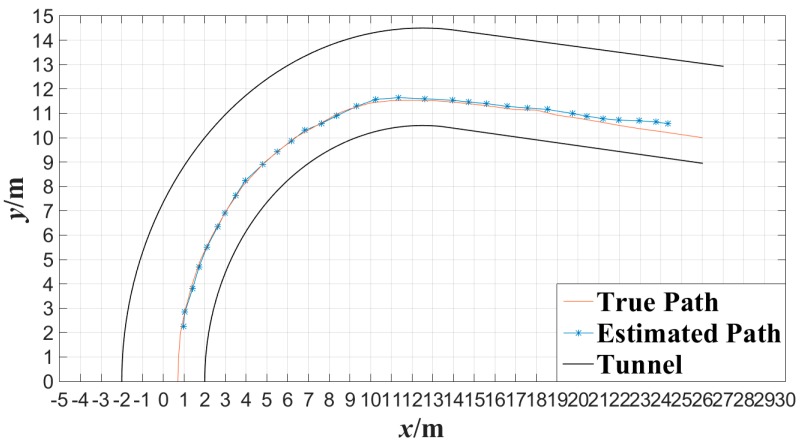
Trajectory of curved line tracked with the miner’s lamp video collaborative localization.

In order to evaluate the performance of the proposed algorithm under the condition that miner moves continuously, cameras *C*_1_, *C*_2_, *C*_3_, *C*_4_, *C*_5_ and *C*_6_ were also applied to detect the movement of the tester in the tunnel. The tester walked along a 90° broken line and a curved line in the tunnel at a normal pace, respectively. Figure 16 shows the tester’s actual motion trajectory along the 90° broken line as well as the estimated motion trajectory with the proposed algorithm at interval of 0.5 s. Figure 16 shows the tester’s actual motion trajectory along the curved tunnel as well as the estimated motion trajectory with the proposed algorithm at interval of 0.5 s. In Figure 16 and Figure 17, the motion trajectory estimated is obtained by connecting two adjacent tester’s locations with line segments. From Figure 16 and Figure 17, it can be observed that the motion trajectory of a miner can be estimated accurately by locating the miner continuously with the proposed miner’s lamp video collaborative localization algorithm. By this way, a miner can be tracked along tunnels.

## 5. Conclusions

Wireless multimedia sensor networks (WMSNs) will improve the monitoring performance of underground coal mines significantly. Based on WMSNs deployed in underground coal mine, a miner’s lamp video collaborative localization algorithm was proposed to locate miners in the scene of bifurcated structures of underground tunnels.

(1) To detect a miner’s lamp, it is proposed to apply background difference method to get the difference value between the background image of underground tunnels and the current image of the video sequences collected.
(2) A least squares method is proposed to find the optimal intersection which solves the problem that multiple straight lines between the positions of cameras and their corresponding mapping points generally cannot intersect at one point.
(3) The experimental results in a corridor indicate that the average error of the altitude localization is 3.7 cm, and the average error of the plane localization is about 10 cm with the proposed algorithm. The experimental results in an underground tunnel indicate that the average error of the altitude localization for up-right and squat postures of the tester are 4.7 cm and 3.9 cm respectively, and the average error of the plane localization for up-right posture of the tester is 27.4 cm with the proposed algorithm.
